# Clinical study on anorexia in patients with terminal cancer

**DOI:** 10.20407/fmj.2024-002

**Published:** 2024-08-28

**Authors:** Miyo Murai, Masanobu Usui, Akihiro Ito, Akihiko Futamura, Kazuki Imai

**Affiliations:** Department of Surgery and Palliative Medicine, Fujita Health University, School of Medicine, Toyoake, Aichi, Japan

**Keywords:** Patients with terminal cancer, Anorexia, Clinical symptoms

## Abstract

**Objectives:**

Patients with terminal cancer tend to have an increased frequency of a variety of clinical symptoms, including anorexia, at 1 month of life expectancy. This study examined clinical symptoms affecting anorexia in patients with terminal cancer.

**Methods:**

Of 1068 patients with terminal cancer who died and were discharged from our hospital between April 2014 and March 2016, we included 471 patients whose clinical symptoms could be subjectively assessed within 4 weeks before death.

Patients were evaluated subjectively on a scale of 0 to 10 (11-point scale) using the Numerical Rating Scale (NRS) once a week for nine major clinical symptoms: (1) pain, (2) general fatigue, (3) dyspnea, (4) depressed mood, (5) anorexia, (6) insomnia, (7) nausea, (8) constipation, and (9) dry mouth. Primary data within 4 weeks prior to death were used for analysis, and Spearman’s rank correlation was used to examine clinical symptoms affecting anorexia.

**Results:**

Spearman’s rank correlation coefficients between anorexia and the following clinical symptoms were: pain, 0.186; general fatigue, 0.414; dyspnea, 0.15; depressed mood, 0.287; insomnia, 0.327; nausea, 0.297; constipation, 0.215; and dry mouth, 0.204. General fatigue was positively correlated with anorexia.

**Conclusions:**

General fatigue may influence anorexia in patients with terminal cancer.

## Introduction

Anorexia is observed in almost all patients with terminal cancer and is one of the factors that can afflict patients.^1^ Anorexia can be induced by the tumor itself or by physical distress as a side effect of treatment (dysgeusia, nausea, diarrhea, pain, dyspnea, etc.) or psychological distress such as depression and sleep disturbance. The frequency of various clinical symptoms, including anorexia, tends to increase in patients with terminal cancer as they approach their last month of life expectancy.^2^ However, there has been little examination of the factors that lead to anorexia in relation to clinical symptoms. Thus, we examined clinical symptoms affecting anorexia in patients with terminal cancer in this study.

## Methods

### Participants

Of 1068 patients with terminal cancer who were discharged or died after being admitted to Fujita Medical University Hospital for palliative treatment between April 2014 and March 2016, 471 patients who could be subjectively assessed by Fujita-style comprehensive assessment of clinical symptoms within 4 weeks prior to death were included in this retrospective study. Blanket consent was obtained for all patients upon admission, and this study was approved by the Fujita Medical School Medical Research Ethics Review Committee (HM 16-401).

### Methods

Patients were subjectively rated on an 11-point scale from 0 to 10 using the Numerical Rating Scale (NRS) on nine major clinical symptoms: (1) pain, (2) general fatigue, (3) dyspnea, (4) depressed mood, (5) anorexia, (6) insomnia, (7) nausea, (8) constipation, and (9) dry mouth at admission and weekly after admission. This clinical symptom assessment tool is a revised version of the Edmonton Symptom Assessment System and has been used since the start of our course as the Fujita-style comprehensive assessment of clinical symptoms.^3,4^ In the present study, the results of the first NRS evaluation performed within 4 weeks prior to death were used as the data when more than one NRS evaluation had been performed on a single patient.

First, the median NRS, interquartile range, and percentage of patients scoring ≥7 for each clinical condition were determined. The Japanese Society for Palliative Medicine’s Pharmacological Treatment Recommendations for Cancer Pain (2020 edition) classifies pain intensity NRS 1–3 as mild, 4–6 as moderate, and 7–10 as severe, and accordingly, a score ≥7 was defined as having severe symptoms. Next, the percentage of each clinical condition with an NRS score ≥7 was examined by time of evaluation (3–4 weeks, 2–3 weeks, 1–2 weeks, and 0–1 week before death), sex, age (divided into two groups by younger or older than 65 years), and primary site (lung, digestive organs, hepatobiliary, and others). Spearman’s rank correlation was used to assess the association between each clinical symptom, and the clinical symptoms that correlated with anorexia were identified.

## Results

Table 1 shows the patient background. Of the 471 participants, 264 were males and 207 were females. The average age was 69.3±11.4 years. The primary site was the lung in 78 (16.6%), stomach in 75 (15.9%), large intestine in 68 (14.4%), pancreas in 62 (13.1%), liver in 47 (10.0%), gynecological and urological organs in 27 (5.7%), esophagus in 22 (4.7%), breast in 21 (4.5%), biliary tract in 19 (4.0%), head and neck in 12 (2.5%), small intestine in 3 (0.6%), hematopoietic organs and thymus in 1 (0.2%), and unknown in 8 (1.7%). NRS was assessed at 3–4 weeks before death in 172 patients (36.5%), 2–3 weeks before death in 122 patients (25.9%), 1–2 weeks before death in 106 patients (22.5%), 0–1 week before death in 71 patients (15.1%).

Table 2 shows the median (interquartile range) NRS scores for each clinical symptom and the percentage of patients with an NRS score ≥7. The following were also reported: anorexia, 4 points (1–7) (30.6%); pain, 2 points (0–4) (10.2%); general fatigue, 3 points (1–5) (18.5%); dyspnea, 0 points (0–3) (6.8%); depressed mood, 1 point (0–4) (10.8%); insomnia, 0 points (0–3) (7.6%); nausea, 0 points (0–2) (5.5%); constipation, 0 points (0–3) (7.6%); and dry mouth, 0 points (0–5) (14.0%).

Table 3 shows the percentage of each clinical symptom with an NRS score ≥7 by time of evaluation, sex, age, and primary site. By time of evaluation, at any evaluation time anorexia remained at a high percentage of approximately 30%. Pain and general fatigue increased over time. There was a trend toward increased rates of dyspnea, depressed mood, and insomnia in the 0–1 week prior to death. By sex, both male and female patients showed the highest percentages for anorexia at 30.3% and 30.9%, respectively. Men were more likely to report general fatigue, and women were more likely to report depressed mood, while there was little difference between men and women for the other symptoms. By age, the highest percentages for anorexia were 33.1% for those <65 years old and 29.5% for those ≥65 years old. The percentages were higher for all symptoms for those <65 years of age than that for >65 years of age. By primary site, the proportions of patients with anorexia were high across all sites, with 32.1% for lung, 30.4% for gastrointestinal, 27.3% for hepatobiliary and pancreatic, and 34.0% for other sites. Dyspnea occurrence rates were high in patients with the lung as the primary site (14.1%), whereas among patients with hepatobiliary and pancreatic cancer, symptoms tended to be lower in the 2%–3% range for dyspnea, nausea, and constipation.

Table 4 shows the results of Spearman’s rank correlation between each clinical symptom. Spearman’s rank correlation coefficients between anorexia and other symptoms were as follows: 0.186 for pain, 0.414 for general fatigue, 0.15 for dyspnea, 0.287 for depressed mood, 0.327 for insomnia, 0.297 for nausea, 0.215 for constipation, and 0.204 for dry mouth. A positive correlation was found between anorexia and general fatigue. Positive correlations were also found between depressed mood and the following symptoms: general fatigue, 0.534; insomnia, 0.534; dyspnea, 0.418; between constipation and dry mouth, 0.45; and insomnia and dry mouth, 0.411. Significant associations were observed between anorexia and dyspnea with p=0.001. For all other symptoms, the p-values were <0.001.

## Discussion

Anorexia is observed in almost all patients with terminal cancer and is one of the factors that can afflict patients.^1^ Conditions that caused decreased oral intake in patients with terminal cancer and an estimated life expectancy ≤1 month include (1) situational factors (odor, taste, unrelieved pain, etc.), (2) medical factors (stomatitis, infection, hypercalcemia, hyperglycemia, low nutrition, constipation, gastrointestinal obstruction, gastroduodenal ulcer, gastritis, drug therapy, dyspepsia syndrome, and intracranial hypertension), and (3) psychological factors (depression and anxiety).^5^ Patients with terminal cancer and a life expectancy ≤1 month are considered to be in a state of refractory cachexia based on the European Palliative Care Research Collaborative classification.^6^ Cancer cachexia is defined as a complex syndrome characterized by a marked loss of skeletal muscle mass (with or without loss of fat mass) that is difficult to correct with conventional nutritional therapy and results in progressive functional disability. In the state of cancer cachexia, activation of inflammatory cytokines plays a central role and has been shown to be deeply involved in various metabolic abnormalities and anorexia.^7^

Tsuneto reported the incidence of physical symptoms in patients with terminal cancer over time and found an increasing trend in clinical symptoms such as general fatigue, anorexia, constipation, and insomnia beginning around 1 month of life expectancy.^2^ In this study, anorexia accounted for the highest percentage of patients with ≥7 NRS points at 30.6% in the last month of life and remained high in the 30% range over time, indicating that patients with terminal cancer suffer from anorexia during their last month of life. In addition, anorexia was considered the most distressing of the major clinical symptoms for patients, regardless of sex, age, or primary site. General fatigue was positively correlated with anorexia (r=0.414). The strength of the association between anorexia and general fatigue at the time of evaluation (3–4 weeks, 2–3 weeks, 1–2 weeks, and 0–1 weeks before death) showed no difference (r=0.4367, 0.3593, 0.4236, and 0.4243, respectively, all p<0.001). In this study, we considered that general fatigue may be the most influential factor in anorexia, and because anorexia is elicited by physical and mental distress, as mentioned earlier, we will pay attention to other clinical symptoms.

The 2012 Survey and Research Project on Nutritional Management of Cancer Patients at the End of Life reported that oral intake is important to 95.7% of patients and 93.5% of their families and that it is important for patients and their families to be able to maintain oral intake for nutritional management as much as possible even at the end of life.^8^ Therefore, when anorexia appears and the patient is unable to eat, both the patient and family become aware of death. Amano et al. focused on eating-related distress in patients with advanced cancer and their families, and reported that multidisciplinary care that also focuses on psychosocial distress may alleviate the eating-related distress of patients’ families.^9^

This study has some limitations. First, it is a single-center, retrospective study. Therefore, the results of this study may not apply to other institutions. Second, the analysis was performed using only the results of subjective evaluation by the patients and not objective data such as blood tests. Although it has the advantage of being less invasive, considering the increasing number of older patients with dementia in the current population, it may be more appropriate to objectively evaluate them using the Integrated Palliative Care Outcome Scale for staff or other methods.^10^

Anorexia is a highly distressing clinical symptom for patients with terminal cancer, and the clinical symptom affecting anorexia the most could be general fatigue.

## Figures and Tables

**Table1:** Patient background

Number of patients	471
Sex (male/female)	264/207
Age, year (mean±SD^a^)	69.3±11.4
Origin n (%)	
lung	78 (16.6)
gastric	75 (15.9)
colorectal	68 (14.4)
pancreas	62 (13.1)
liver	47 (10.0)
uterus/adnexa	27 (5.7)
renal/urinary tract	27 (5.7)
oesophageal	22 (4.7)
breast	21 (4.5)
biliary tract	19 (4.0)
head and neck	12 (2.5)
small intestine	3 (0.6)
hematopoietic organs	1 (0.2)
thymus	1 (0.2)
unknown	8 (1.7)
Evaluation week n (%)	
before death 3–4W	172 (36.5)
before death 2–3W	122 (25.9)
before death 1–2W	106 (22.5)
before death 0–1W	71 (15.1)

^a^, SD, Standard deviation

**Table2:** NRS median, interquartile range, and percentage scoring 7 or higher

	n	median (IQR^a^)	7≤NRS^b^
anorexia	471	4 (1–7)	144 (30.6%)
pain	471	2 (0–4)	48 (10.2%)
general fatigue	471	3 (1–5)	87 (18.5%)
dyspnea	471	0 (0–3)	32 (6.8%)
depression	471	1 (0–4)	51 (10.8%)
insomnia	471	0 (0–3)	36 (7.6%)
nausea	471	0 (0–2)	26 (5.5%)
constipation	471	0 (0–3)	36 (7.6%)
dry mouth	471	0 (0–5)	66 (14.0%)

^a^, NRS, Numerical Rating Scale; IQR, interquartile range

**Table3:** Percentage of NRS scores 7 or higher for each clinical symptom by time of evaluation, sex, age, and primary site

	7≤NRS^a^									
Evaluation week	n	anorexia	pain	general fatigue	dyspnea	depression	insomnia	nausea	constipation	dry mouth
before death 3–4W	172	30.2%	7.0%	13.4%	5.2%	10.5%	6.4%	5.2%	12.2%	16.9%
before death 2–3W	122	32.8%	9.8%	15.6%	4.1%	6.6%	0.8%	6.6%	7.4%	9.8%
before death 1–2W	106	29.2%	12.3%	20.8%	4.7%	11.3%	9.4%	4.7%	2.8%	14.2%
before death 0–1W	71	29.6%	14.1%	32.4%	18.3%	18.3%	19.7%	5.6%	4.2%	14.1%
Sex										
male	264	30.3%	9.8%	19.3%	6.8%	9.5%	8.0%	5.3%	7.6%	14.0%
female	207	30.9%	10.6%	17.4%	6.8%	12.6%	7.2%	5.8%	7.7%	14.0%
Age										
<65	139	33.1%	13.7%	23.0%	7.9%	19.4%	10.1%	7.2%	8.6%	14.4%
65≤	332	29.5%	8.7%	16.6%	6.3%	7.2%	6.6%	4.8%	7.2%	13.9%
Origin										
lung	78	32.1%	10.3%	15.4%	14.1%	11.5%	9.0%	6.4%	9.0%	14.1%
digestive organ	168	30.4%	8.9%	19.6%	6.5%	11.9%	6.5%	5.4%	9.5%	14.9%
Hepatobiliary pancreas	128	27.3%	9.4%	21.1%	2.3%	8.6%	7.8%	3.1%	3.1%	9.4%
others	97	34.0%	13.4%	15.5%	7.2%	11.3%	8.2%	8.2%	9.3%	10.3%

^a^, NRS, Numerical Rating Scale

**Table4:** Spearman’s rank correlations between clinical symptoms

	anorexia	pain	general fatigue	dyspnea	depression	insomnia	nausea	constipation	dry mouth
anorexia	—	—	—	—	—	—	—	—	—
pain	0.186	—	—	—	—	—	—	—	—
general fatigue	0.414	0.355	—	—	—	—	—	—	—
dyspnea	0.15^a^	0.194	0.338	—	—	—	—	—	—
depression	0.287	0.357	0.534	0.418	—	—	—	—	—
insomnia	0.327	0.312	0.397	0.374	0.534	—	—	—	—
nausea	0.297	0.217	0.309	0.222	0.252	0.331	—	—	—
constipation	0.215	0.268	0.275	0.224	0.304	0.295	0.319	—	—
dry mouth	0.204	0.162	0.259	0.328	0.364	0.411	0.259	0.45	—

^a^, p=0.001, all others p<0.001

## Supplementary Material

PDF-Japanese
