# Supplementary material for: Clinical study on anorexia in patients with terminal cancer
Source: Fujita Med J. 2024 Aug 28;10(4):94–7. doi: 10.20407/fmj.2024-002 (PMC11528327; doi:10.20407/fmj.2024-002)
Supplement: Supplementary file 1 — PDF-Japanese [file fmj-10-094-s001.pdf]

終末期がん患者の食欲不振に関する臨床的研究

ランニングタイトル：終末期がん患者の食欲不振に関する臨床的研究

Miyo Murai, MD, PhD, Usui Masanobu, MD, PhD, Ito Akihiro, MD, PhD, Futamura Akihiko , PhD, Imai Kazuki, PhD

Department of Surgery and Palliative Medicine, Fujita Health University, School of Medicine, Toyoake, Aichi, Japan

Original Article

Corresponding author: Usui Masanobu, MD, PhD

Department of Surgery and Palliative Medicine, Fujita Health University,
School of Medicine, 1-98 Dengakugakubo, Kutsukakecho, Toyoake, Aichi
470-1192, Japan

Tel: 0562-93-2111

E-mail: masanobu.usui@fujita-hu.ac.jp

Abstract

目的：終末期がん患者は、余命 1 か月頃から食欲不振をはじめとする多彩な臨床症状の出現頻度が増加する傾向がある。本研究では、終末期がん患者の食欲不振に影響を及ぼす臨床症状について検討した。

方法：2014 年 4 月から 2016 年 3 月までに当院を死亡退院した終末期がん患者 1068 例のうち、死亡前 4 週以内で臨床症状の主観的評価が可能であった 471 例を対象とした。

方法：患者は、週 1 回定期的に、①疼痛、②全身倦怠感、③呼吸困難、④気分の落込み、⑤食欲不振、⑥不眠、⑦吐気、⑧便秘、⑨口渇の 9 項目の主要臨床症状について Numerical Rating Scale(NRS)を用い 0〜10 の 11 段階で主観的評価を実施した。本研究では死亡前 4 週以内の初回データを解析に用い、スピアマンの順位相関を用いて食欲不振に影響を及ぼす臨床症状を検討した。

結果：スピアマンの順位相関係数は、食欲不振と疼痛 0.186、食欲不振と全身倦怠感 0.414、食欲不振と呼吸困難 0.15、食欲不振と気分の落込み 0.287、食欲不振と不眠 0.327、食欲不振と吐気 0.297、食欲不振と便秘 0.215、食欲不振と口渇 0.204 となり全身倦怠感が食欲不振と正の相関を認めた。

結論：全身倦怠感は終末期がん患者の食欲不振に影響を及ぼすことが考えられた。

key words: 終末期がん患者、食欲不振、臨床症状

序論

食欲不振は終末期がん患者ではほぼ全例で認められており[1)]、患者を悩ませる因子のひとつである。食欲不振は、腫瘍そのものや、治療の副作用としての身体的苦痛（味覚障害、悪心、下痢、痛み、呼吸困難など）や抑うつ・睡眠障害などの精神的苦痛によって惹起される。終末期がん患者は余命 1 か月頃から、食欲不振をはじめとする多彩な臨床症状の出現頻度が増加する傾向がみられている[2)]。しかし、これまで臨床症状と関連して総合的に食欲不振につながる因子の検討はほとんどされていない。そこで本研究では、終末期がん患者の食欲不振に影響を及ぼす臨床症状について検討した。

方法

対象：2014 年 4 月から 2016 年 3 月までに藤田医科大学病院に入院し緩和治療を受けた後、死亡退院した終末期がん患者 1068 例のうち、死亡前 4 週以内で藤田式臨床症状総合評価の主観的評価が可能であった 471 例を対象とし、後方視的に調査した。全患者には入院時に包括的同意を得ており、本研究は藤田医科大学 医学研究倫理審査委員会(HM 16-401)の承認を得ている。

方法：患者は、入院時と入院後は毎週定期的に、①疼痛、②全身倦怠感、③呼吸困難、④気分の落込み、⑤食欲不振、⑥不眠、⑦吐気、⑧便秘、⑨口渇の 9 項目の主要臨床症状につ

いて Numerical Rating Scale(NRS)を用い 0～10 の 11 段階で主観的評価を実施した。この臨床症状のアセスメントツールは、エドモントン症状評価システム[3]を改訂したもので、藤田式臨床症状総合評価として当講座開講以来使用している[4]。本研究では、死亡前 4 週以内に実施された NRS 評価がひとりの患者につき複数回実施されていた場合、その初回評価の結果をデータベースとして用いた。

まず、各臨床症状の NRS の中央値、四分位範囲、7 点以上の割合を求めた。日本緩和医療学会の「がん疼痛の薬物療法に関するガイドライン（2020 年版）」では、痛みの強さを NRS 1-3 を軽度、4-6 を中等度、7-10 を高度と定めており、これに準じて 7 点以上を高度な症状を有するとした。次に、評価時期別（死亡前 3-4 週、2-3 週、1-2 週、0-1 週）、男女別、年齢別（65 歳で 2 群に分類）、原発部位別（肺、消化器、肝胆膵、その他で検討）で、各臨床症状の NRS7 点以上の割合を求めた。そして、各臨床症状間でスピアマンの順位相関を実施し、食欲不振と相関を認める臨床症状を抽出した。

結果

表 1 に患者背景を示す。471 名の内訳は、男性 264 名、女性 207 名。平均年齢は、69.3±11.4 歳。原発部位は、肺 78 例(16.6%)、胃 75 例(15.9%)、大腸 68 例(14.4%)、膵 62 例(13.1%)、肝 47 例(10.0%)、婦人科・泌尿器 各 27 例(5.7%)、食道 22 例(4.7%)、乳腺 21 例(4.5%)、胆道 19 例(4.0%)、頭頸部 12 例(2.5%)、小腸 3 例(0.6%)、造血器・胸腺各 1 例(0.2%)、原発不明 8 例(1.7%)。NRS の評価時期は、死亡前 3～4 週 172 例(36.5%)、死亡前 2～3 週 122 例(25.9%)、死亡前 1～2 週 106 例(22.5%)、死亡前 0-1 週 71 例(15.1%)であった。

表 2 に各臨床症状の NRS の中央値（四分位範囲)、NRS が 7 点以上の割合を示す。それぞれ、食慾不振：4 点(1-7) 30.6%、疼痛：2 点(0-4) 10.2%、全身倦怠感：3 点(1-5) 18.5%、呼吸困難：0 点(0-3) 6.8%、気分の落ち込み：1 点(0-4) 10.8%、不眠：0 点(0-3) 7.6%、吐気：0 点(0-2) 5.5%、便秘：0 点(0-3) 7.6%、口渇：0 点(0-5) 14.0%であった。

表 3 に評価時期別、男女別、年齢別、原発部位別での各臨床症状の NRS 7 点以上の割合を示す。評価時期別：食欲不振では全て 30%前後の高い割合で推移していた。疼痛と全身倦怠感では経時的に増加していた。呼吸困難、気分の落ち込み、不眠では死亡前 0-1 週で割合が増加する傾向が見られた。男女別：男女とも食欲不振でそれぞれ 30.3%、30.9%と最も高い割合を示した。全身倦怠感では男性、気分の落ち込みでは女性で割合が高く、その他の症状では男女差はほとんどみられなかった。年齢別：食欲不振では 65 歳未満で 33.1%、65 歳以上で 29.5%と最も高い割合を示した。65 歳未満の方が全ての症状で割合は高かった。原発部位別：肺、消化器、肝胆膵、その他とも食慾不振でそれぞれ 32.1%、30.4%、27.3%、34.0%と高い割合を示した。肺は呼吸困難で 14.1%と高く、肝胆膵は、呼吸困難、吐気、便秘で 2-3%台と低い傾向にあった。

表 4 に各臨床症状間でスピアマンの順位相関を実施した結果を示す。スピアマンの順位相関係数は、食欲不振と疼痛 0.186、食欲不振と全身倦怠感 0.414、食欲不振と呼吸困難

0.15、食欲不振と気分の落込み 0.287、食欲不振と不眠 0.327、食欲不振と吐気 0.297、食欲不振と便秘 0.215、食欲不振と口渇 0.204 で、食慾不振と全身倦怠感との間に正の相関を認めた。気分の落ち込みと全身倦怠感 0.534、気分の落ち込みと不眠 0.534、気分の落ち込みと呼吸困難 0.418、便秘と口渇 0.45､、不眠と口渇 0.411 との間にもそれぞれ正の相関を認めた。なお、有意差は食欲不振と呼吸困難で p=0.001 であり、それ以外は全て p<0.001 であった。

考察

食欲不振は終末期がん患者ではほぼ全例で認められていて[1]、患者を悩ませる因子のひとつである。推定余命 1 カ月以内の終末期がん患者の経口摂取の低下をきたす病態として、①状況要因（におい、味、緩和されていない苦痛など）、②医学的要因（口内炎、感染症、高カルシウム血症、高血糖、低栄養、便秘、消化管閉塞、胃・十二指腸潰瘍、胃炎、薬物、胃拡張不全症候群、頭蓋内圧亢進）、③精神的要因（抑うつ、不安）が挙げられる[5]。余命 1 カ月以内の終末期がん患者は、EPCRC(European Palliative Care Research Collaborative) の分類に基づくと不応性悪液質の状態に陥っていると考えられる[6]。がん悪液質とは、通常の栄養療法で改善することは困難で、進行性に機能障害をもたらす、骨格筋量の著しい減少（脂肪量の減少の有無にかかわらず）を特徴とする複合的な症候群、と定義される。がん悪液質の状態では、炎症性サイトカインの活性化が中心的な役割をはたし、さまざまな代謝異常や食欲不振に深く関与していることが明らかとなっている[7]。

恒藤は、終末期がん患者の身体症状の出現頻度を経時的に報告し、余命 1 か月頃から、全身倦怠感、食欲不振、便秘、不眠などの多彩な臨床症状が出現する頻度が増加する傾向が見られたことを報告している[2]。本研究では、余命 1 カ月で NRS7 点以上の割合が食欲不振で 30.6％と最も高く、かつ、経時的にみても 30％台の高い数値で推移していることから、終末期がん患者は余命 1 カ月の間、食欲不振に悩まされていることが示唆された。加えて食欲不振は、性別、年齢、原発部位に関係なく主要な臨床症状の中で最も患者を苦しめる症状であると考えられた。食欲不振と正の相関関係を認めたのは全身倦怠感であった (r=0.414)。評価時期（死亡前 3-4 週、2-3 週、1-2 週、0-1 週）での食慾不振と全身倦怠感の関連の強さは、それぞれ r=0.4367、0.3593、0.4236、0.4243（全て p<0.001）であることから評価時期でほとんど差がみられない傾向にあった。本研究では、全身倦怠感が食欲不振に最も影響を及ぼす因子である可能性があることが考えられた、食欲不振は先述したように、身体的苦痛や精神的苦痛によって惹起されることから、他の臨床症状にも注意を払っていきたいと考える。

かつて、平成 24 年度の「終末期がん患者の栄養管理に関わる調査研究事業」では、経口摂取の重要度は患者で 95.7%、患者家族で 93.5%で、終末期においても経口摂取による栄養管理を可能な限り維持できることが患者および患者家族にとって大事であると報告されている[8]。それゆえに食欲不振が出現し食べられなくなると患者も家族も死を意識するよう

になる。天野らは、進行がん患者と家族の食に関する苦悩（eating-related distress）に注目し、心理社会的苦痛にも焦点を当てた多職種での集学的なケアを実施することが患者家族の食に関する苦悩を軽減できるかもしれないと報告している[9)]。

　この研究の限界としては以下の事が挙げられる。第一に、単施設における後方視的研究である点である。そのため本研究の結果は他施設にあてはまらないかもしれない。第二に、血液検査等の客観的データは用いず患者の主観的評価の結果のみで解析を行った点である。侵襲が少ないという利点はあるが、高齢で認知症の患者が増加しつつある現代社会を考慮すると、IPOS(Integrated Palliative care Outcome Scale)[10)] スタッフ用などを用いて客観的に評価する方が適切かもしれない。

　食欲不振は、終末期がん患者を最も苦しめる臨床症状であり、食欲不振に影響を及ぼす臨床症状は全身倦怠感である可能性があることが考えられた。

利益相反

　本研究に関する利益相反はない。

謝辞

　統計に関して、藤田医科大学 橋渡し研究統括本部 橋渡し研究シーズ探索センター 生物統計室　嘉田晃子先生より、貴重なご指導とご助言を賜りました。感謝申し上げます。

引用文献

1. Amano K, Maeda I, Morita, T et al. C-reactive protein, symptoms and activity of daily living in patients with advanced cancer receiving palliative care. J Cachexia Sarcopenia Muscle 2017 ; 8 : 457-65.
2.Tsunetou A. Saishin kanwa iryogaku(Latest palliative medicine). Osaka：Saishinigakusha　；1999:18-21(in Japanese).
3. Bruera E, Kuehn N, Miller MJ, Selmser P, Macmillan K. The Edmonton Symptom Assessment System (ESAS): a simple method for the assessment of palliative care patients. J Palliat Care 1991; 7:6-9.
4.Murai M, Higashiguchi T, Futamura A, Ohara H, Tsuzuki N, Itani Y, Kaneko T, Chihara T, Shimpo K, Nakayama N. Interleukin-8 and clinical symptoms can be prognostic indicators for advanced cancer patients with cachexia. Fujita Med J 2020; 6: 117-21.
5. Japanese Society for Palliative Medicine. Clinical guidelines for infusion therapy in advanced cancer patients. 2013 ed. Tokyo: Kanehara & Co.; 2013.
6. Fearon K, Strasser F, Anker SD, et al. Definition and classification of cancer cachexia: an international consensus. Lancet Oncol 2011; 12: 489–95.
7. Evans WJ, Morley JE, Argilés J, et al.　Cachexia: a new definition. Clin Nutr 2008；27:793–9.
8. Fujita Academy. Shumakki gan kanja no eiyo kanri ni kakawaru chosa kenkyu jigyau hokokusho(Investigation and Research Project Report on Nutritional Management for End-stage Cancer Patients in the Fiscal Year 2012 Elderly Health Promotion Program）. Fujita Academy; 2013.
9.Amano K, Hopkinson J, Baracos V. Psychological symptoms of illness and emotional distress in advanced cancer cachexia. Curr Opin Clin Nutr Metab Care 2022; 25: 167-72.
10. Sakurai H, Miyashita M, Imai K, Miyamoto S, Otani H, Oishi A, Kizawa Y, Matsushima E. Validation of the Integrated Palliative care Outcome Scale (IPOS) - Japanese Version. Jpn J Clin Oncol 2019; 49: 257-62.

表１　患者背景

ＳＤ：標準偏差

表２　各臨床症状の NRS の中央値、四分位範囲、7 点以上の割合

NRS：Numerical Rating Scale、IQR:interquartile range

表３　評価時期別、男女別、年齢別、原発部位別での各臨床症状の NRS 7 点以上の割合

NRS：Numerical Rating Scale

表４　各臨床症状間でのスピアマンの順位相関

a）$p=0.001$、それ以外は全て $p<0.001$

Table 1

Number of patients	471
Sex(male/female)	264/207
Age,year (mean±SD)	69.3±11.4
Origin n(%)	
lung	78(16.6)
gastric	75(15.9)
colorectal	68(14.4)
pancreas	62(13.1)
liver	47(10.0)
uterus/adnexa	27(5.7)
renal/urinary tract	27(5.7)
oesophageal	22(4.7)
breast	21(4.5)
biliary tract	19(4.0)
head and neck	12(2.5)
small intestine	3(0.6)
hematopoietic organs	1(0.2)
thymus	1(0.2)
unknown	8(1.7)
Evaluation week n(%)	
before death 3-4W	172(36.5)
before death 2-3W	122(25.9)
before death 1-2W	106(22.5)
before death 0-1W	71(15.1)

Table 2

	n	median(IQR)	7≦NRS
anorexia	471	4(1-7)	144(30.6%)
pain	471	2(0-4)	48(10.2%)
general fatigue	471	3(1-5)	87(18.5%)
dyspnea	471	0(0-3)	32(6.8%)
depression	471	1(0-4)	51(10.8%)
insomnia	471	0(0-3)	36(7.6%)
nausea	471	0(0-2)	26(5.5%)
constipation	471	0(0-3)	36(7.6%)
dry mouth	471	0(0-5)	66(14.0%)

Table 3

		7≦NRS								
Evaluation week	n	anorexia	pain	general fatigue	dyspnea	depression	insomnia	nausea	constipation	dry mouth
before death 3-4W	172	30.2%	7.0%	13.4%	5.2%	10.5%	6.4%	5.2%	12.2%	16.9%
before death 2-3W	122	32.8%	9.8%	15.6%	4.1%	6.6%	0.8%	6.6%	7.4%	9.8%
before death 1-2W	106	29.2%	12.3%	20.8%	4.7%	11.3%	9.4%	4.7%	2.8%	14.2%
before death 0-1W	71	29.6%	14.1%	32.4%	18.3%	18.3%	19.7%	5.6%	4.2%	14.1%
Sex										
male	264	30.3%	9.8%	19.3%	6.8%	9.5%	8.0%	5.3%	7.6%	14.0%
female	207	30.9%	10.6%	17.4%	6.8%	12.6%	7.2%	5.8%	7.7%	14.0%
Age										
<65	139	33.1%	13.7%	23.0%	7.9%	19.4%	10.1%	7.2%	8.6%	14.4%
65≦	332	29.5%	8.7%	16.6%	6.3%	7.2%	6.6%	4.8%	7.2%	13.9%
Origin										
lung	78	32.1%	10.3%	15.4%	14.1%	11.5%	9.0%	6.4%	9.0%	14.1%
digestive organ	168	30.4%	8.9%	19.6%	6.5%	11.9%	6.5%	5.4%	9.5%	14.9%
Hepatobiliary pancreas	128	27.3%	9.4%	21.1%	2.3%	8.6%	7.8%	3.1%	3.1%	9.4%
others	97	34.0%	13.4%	15.5%	7.2%	11.3%	8.2%	8.2%	9.3%	10.3%

Table 4

	anorexia	pain	general fatigue	dyspnea	depression	insomnia	nausea	constipation	dry mouth
anorexia	-	-	-	-	-	-	-	-	-
pain	0.186	-	-	-	-	-	-	-	-
general fatigue	0.414	0.355	-	-	-	-	-	-	-
dyspnea	0.15[a]	0.194	0.338	-	-	-	-	-	-
depression	0.287	0.357	0.534	0.418	-	-	-	-	-
insomnia	0.327	0.312	0.397	0.374	0.534	-	-	-	-
nausea	0.297	0.217	0.309	0.222	0.252	0.331	-	-	-
constipation	0.215	0.268	0.275	0.224	0.304	0.295	0.319	-	-
dry mouth	0.204	0.162	0.259	0.328	0.364	0.411	0.259	0.45	-
